# Predictors of Prolonged Hospitalization in Pyogenic Tenosynovitis of the Hand: A Retrospective Cohort Study Focused on Length of Stay

**DOI:** 10.3390/medicina62030534

**Published:** 2026-03-13

**Authors:** Cristian-Sorin Hariga, Florin-Vlad Hodea, Vladut-Alin Ratoiu, Eliza-Maria Bordeanu-Diaconescu, Madalina-Olivia Radu-Adamesteanu, Razvan Nicolae Teodoreanu, Cristian-Radu Jecan, Andreea Grosu-Bularda

**Affiliations:** 1 Department 11, Discipline Plastic and Reconstructive Surgery, Bucharest Clinical Emergency Hospital, University of Medicine and Pharmacy Carol Davila, 050474 Bucharest, Romania; cristian.hariga@umfcd.ro (C.-S.H.); madalina.adamesteanu@umfcd.ro (M.-O.R.-A.); razvan.teodoreanu@umfcd.ro (R.N.T.); cristian.jecan@umfcd.ro (C.-R.J.); andreea.grosu-bularda@umfcd.ro (A.G.-B.); 2 Clinic of Plastic Surgery, Aesthetic and Reconstructive Microsurgery, Emergency Clinical Hospital Bucharest, 050474 Bucharest, Romania; eliza.diaconescu@umfcd.ro

**Keywords:** septic tenosynovitis, hand infections, length of stay, comorbidities, immunosuppression, healthcare resource

## Abstract

*Background and Objectives:* Septic tenosynovitis of the hand remains a surgical emergency associated with significant morbidity and healthcare resource utilization. While prior studies have focused primarily on diagnostic features, microbiology, and functional outcomes, factors influencing prolonged hospitalization remain insufficiently characterized. Length of stay (LOS) represents a pragmatic, patient- and system-centered outcome that may reflect disease burden and treatment complexity. To identify clinical, demographic, and treatment-related factors associated with prolonged hospitalization in patients treated surgically for septic tenosynovitis of the hand, using LOS ≥ 6 days as a clinically meaningful threshold. *Materials and methods*: A retrospective cross-sectional study was conducted including 38 adult patients treated surgically for acute septic tenosynovitis of the hand at a tertiary referral center between 1 January 2020 and 31 December 2024. Demographic variables, comorbidities, immunosuppressive status, mechanism of injury, anatomical involvement, time to presentation, microbiological findings, number of surgical procedures, inpatient LOS, and outpatient follow-up visits were analyzed. *Results*: The mean age was 53.2 ± 15.8 years, and the mean LOS was 7.5 ± 3.7 days (range, 2–17 days). Twenty-seven patients (71.1%) experienced an LOS ≥ 6 days. The presence of comorbidities (*p* = 0.0026) and immunosuppressive status (*p* = 0.0378) were significantly associated with prolonged hospitalization. In contrast, age, sex, time to presentation, and microbiological culture positivity, were not significantly associated with prolonged LOS. The mean number of outpatient follow-up visits was 2.5 ± 1.9, highlighting an additional post-discharge care burden. *Conclusions*: Prolonged hospitalization in septic tenosynovitis appears to be driven predominantly by patient-related vulnerability, particularly comorbidities and immunosuppression, rather than by anatomical involvement, microbiological profile, or presentation delay alone. LOS may serve as a useful surrogate marker for treatment burden and resource utilization, supporting early identification of high-risk patients and optimization of inpatient care pathways.

## 1. Introduction

Acute septic tenosynovitis of the hand remains a challenging entity in hand surgery, with potentially devastating consequences if diagnosis or treatment are delayed. Although sheaths of flexor tendons are most commonly involved, any tendon sheath of the hand can be affected [1,2]. The closed-space nature of tendon sheaths favors rapid spread of infection, leading to purulence, increased pressure, tendon necrosis, and in severe cases, irreversible functional impairment, stiffness, or even amputation [3,4,5,6,7].

Epidemiologically, pyogenic flexor tenosynovitis accounts for approximately 2.5% to 9.4% of all hand infections, a non-negligible proportion, while actual incidence may be underreported due to misdiagnosis or delayed presentation. Risk factors classically described in the literature include penetrating trauma (e.g., lacerations, punctures, bites), immunosuppression, comorbidities such as diabetes, and intravenous drug use; yet, in a subset of cases, no clear traumatic event can be identified [8,9,10,11,12].

**Figure 1:**
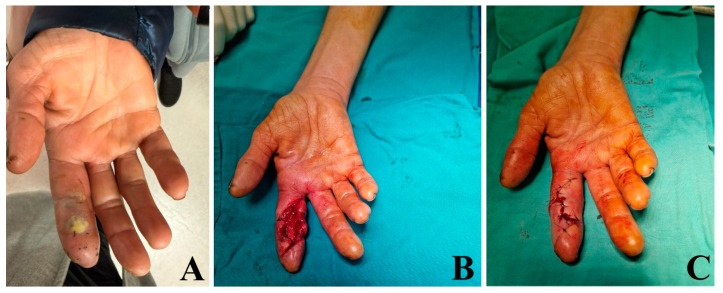
Clinical evolution of a flexor tenosynovitis. (**A**) Clinical aspect of a sting at the level of the middle volar phalanx of the second finger. (**B**) Aspect of the finger after incision, evacuation and debridement. (**C**) Aspect of final aspect after skin closure.

Although pyogenic infections classically involve the flexor tendon sheaths, recent evidence shows that extensor tenosynovitis, once considered rare, is increasingly recognized in clinical practice [13,14]. While flexor sheaths provide a well-defined closed anatomical compartment that facilitates rapid bacterial proliferation, extensor tendons lie in looser areolar tissue, making infection less contained but often more deceptive in its early presentation. Contemporary studies show that extensor involvement may present with more subtle swelling and less characteristic clinical signs, contributing to delayed diagnosis and more advanced disease at the time of surgical exploration [15,16,17]. The aspect of flexor tenosynovitis can be seen in Figure 1, from presentation to debridement and final closure.

Previous studies on pyogenic tenosynovitis have largely focused on diagnostic accuracy, microbiological profiles, or functional outcomes, with LOS often reported as a secondary descriptive variable rather than a primary endpoint. Moreover, existing analyses tend to dichotomize outcomes based on complications or reoperation rates, without specifically interrogating what drives extended hospitalization in patients who survive the acute infectious episode. This leaves an important gap in the literature: identifying which factors meaningfully predispose patients to prolonged inpatient care, and whether these factors are modifiable at the level of timing, surgical strategy, or perioperative management. From a healthcare economics perspective, prolonged hospitalization in acute hand infections carries substantial implications beyond individual patient outcomes. Hand infections disproportionately affect working-age adults, with peak incidence occurring in individuals aged 30–50 years, and the resulting combination of inpatient costs, lost productivity, and extended rehabilitation places a considerable strain on healthcare systems. In the context of increasingly constrained hospital resources and growing pressure toward ambulatory and early discharge models, identifying reliable predictors of prolonged LOS is an essential step toward developing evidence-based perioperative care pathways [18]. Septic tenosynovitis is frequently managed in tertiary centers where bed availability, nursing intensity, and intravenous antibiotic administration are all limiting factors. Early identification of patients at risk for extended hospitalization allows for proactive resource allocation, optimization of bed management, and timely involvement of multidisciplinary support services including infectious disease specialists, physiotherapy, and social work.

The present study aims to address this gap by analyzing a cohort of patients treated surgically for septic flexor tenosynovitis, focusing specifically on determinants associated with a length of stay (LOS) equal to or exceeding six days. This threshold was selected to reflect hospitalization beyond the contemporary average reported in the United States, thereby anchoring the analysis to a clinically meaningful and externally relevant benchmark. Specifically, the 6-day cut-off corresponds to approximately the 75th percentile of LOS in the present cohort (mean 7.5 days, range 2–17 days) and is consistent with the OECD-reported average inpatient durations for surgical procedures in high-income countries [18]. While externally anchored rather than internally data-derived, this threshold identifies patients whose stay substantially exceeds the expected duration for an uncomplicated postoperative course and who represent a disproportionate inpatient resource burden.

By examining demographic variables, comorbidities, presentation characteristics, microbiological findings, and treatment-related factors, this study seeks to identify predictors of prolonged hospitalization and to clarify which elements of septic tenosynovitis management may influence resource utilization and patient recovery.

## 2. Materials and Methods

This 5-year retrospective cross-sectional study included 38 patients admitted to the Clinic of Plastic and Reconstructive Surgery of the Clinical Emergency Hospital Bucharest, Romania, a tertiary care center, between 1 January 2020 and 31 December 2024. The study population consisted of adult patients diagnosed with acute septic tenosynovitis of the hand, both flexor and extensor compartments, admitted through the emergency department.

Inclusion criteria: Adults (≥18 years) with clinically and surgically confirmed septic tenosynovitis, involving any finger or compartment of the hand.

Exclusion criteria: Incomplete clinical or microbiological records, chronic inflammatory conditions of the tendon sheaths, and infections involving anatomical regions other than the hand.

Written informed consent was obtained from all patients upon admission. Data confidentiality followed institutional protocols, with ethical approval granted by the hospital’s Ethics Committee (approval no. 10334/12 December 2025). Data collection was performed using both physical and digital hospital archives. To minimize selection and information bias, data extraction was independently verified by multiple reviewers, and discrepancies were resolved by consensus.

The dataset included demographic, clinical, microbiological and therapeutic variables: age, sex, comorbidities, immunosuppression, mechanism of trauma, anatomical site, time from injury to presentation, presence of tendon rupture, microbiological culture and antibiogram results, number of surgical interventions, need for reintervention, inpatient LOS, and number of outpatient follow-up visits. Immunosuppressive status was defined as the presence of any of the following conditions at time of admission: active malignancy, diabetes mellitus, systemic corticosteroid therapy, biologic immunosuppressive therapy, human immunodeficiency virus infection, or chronic excessive alcohol use.

All patients had the same intervention, using Brunner’s incisions overlying the tenosynovitis of the flexor tendons and italic “S” overlying the extensor tenosynovitis, with evacuation of collections, culturing of discharge, followed by aggressive debridement and washout, and wounds were left open for daily washout until final closing surgery.

Statistical analysis was performed using Microsoft Excel (Microsoft Corp., Redmond, WA, USA) and IBM SPSS Statistics for Windows, Version 29.0 (IBM Corp.; Armonk, NY, USA). Descriptive statistics (means, medians, ranges, and proportions) were used to characterize the cohort. Categorical variables were compared using Fisher’s exact test. The results were subsequently contextualized against existing evidence identified via the databases Web of Science, PubMed, and Google Scholar.

## 3. Results

The study cohort included 38 patients. The mean age was 53.2 ± 15.8 years, with a range from 22 to 89 years. The overall mean length of hospital stay was 7.5 ± 3.7 days, with a wide range spanning from 2 to 17 days. In total, 27 patients (71.1%) experienced an LOS of ≥6 days, while 11 patients (28.9%) were discharged within five days. Regarding sex distribution, 23 patients (60.5%) were male and 15 patients (39.5%) were female. Time from symptom onset or injury to hospital presentation was available for 31 patients. The mean duration until presentation was 9.6 ± 7.4 days, ranging from 1 to 30 days.

Regarding anatomical location, involvement of the index finger (digit II) was most common, observed in 14 patients (36.8%), followed by the middle finger (digit III) in 10 patients (26.3%). The thumb (digit I), ring finger (digit IV), and small finger (digit V) were each involved in four patients (10.5%), while diffuse involvement of the hand without a specific digital localization (H) was recorded in two patients (5.3%). The right hand was affected in 25 patients (65.8%), while 13 (34.2%) had the left hand affected. With respect to tendon compartment involvement, flexor tenosynovitis was identified in 26 patients (68.4%), volar tenosynovitis (involving volar tendon structures without a dominant flexor sheath component) was present in five patients (13.2%), extensor tenosynovitis in six patients (15.8%), and combined flexor and extensor involvement was observed in one patient (2.6%). Tendon rupture was documented intraoperatively in three patients (7.9%), all involving the flexor tendon compartment. Right-hand involvement was more frequent, observed in 25 patients (65.8%), compared to the left hand in 13 patients (34.2%).

A definable mechanism of injury was identified in the majority of cases. Sharp lacerations were most frequent, followed by bite-related injuries, puncture wounds, and others, as seen in Table 1.

LOS was found to be statistically significant in case of presence of comorbidities (*p* = 0.0026) and immunosuppressive status (*p* = 0.0378), while time from symptom onset (*p* = 0.1596), old age (*p* = 1.0), and microbiological status (*p* = 0.491) did not yield any statistically significant relevancy, as seen in Table 2. Among the 14 patients with comorbidities, the most prevalent condition was diabetes mellitus, documented in eight (57%) patients (one patient with type I). Isolated or combined arterial hypertension without other major systemic disease was present in three patients (21.4%), and two patients (14.3%) had other conditions (hypotension with vertiginous syndrome; chronic alcoholism with schizoaffective disorder). Major cardiovascular or multisystem disease (including atrial fibrillation, myocardial infarction, stroke, mixed circulatory insufficiency, or respiratory failure) was present in three patients (21.4%), and chronic renal failure was identified in two patients (14.3%). Note that several patients had overlapping comorbidities. All 14 comorbid patients experienced LOS ≥ 6 days regardless of comorbidity subtype, though the small subgroup sizes precluded statistically meaningful between-subgroup comparisons.

Only 21 patients exhibited bacterial growth from this study. Seventeen patients did not exhibit bacteria growth; since all patients met criteria for clinically and surgically confirmed septic tenosynovitis, non-infectious etiologies represent alternative diagnoses rather than explanations for culture negativity. More plausible explanations include: prior antibiotic exposure before surgical sampling suppressing bacterial growth below culture detection thresholds, biofilm-associated infections reducing recovery on standard media, and the presence of fastidious or slow-growing organisms (e.g., atypical mycobacteria, anaerobes) requiring specialized media or prolonged incubation. Overall, monomicrobial infections accounted for 18 of 21 culture-positive cases (85.7%), while three cases (14.3%) demonstrated polymicrobial growth, with isolated pathogens seen in Table 3. Of note, all bacteria were non-MDR, with the exception of one instance of staphylococcus hominis which was MDR.

All patients underwent two interventions, one for incision-debridement and one for closure, with the exception of two patients, one which required an extra debridement intervention and one patient who required four total debridements and after final closure, he came back with recurrence of tenosynovitis and required amputation. In this case, microbiological analysis revealed that the infection was caused by two pathogens: *Escherichia coli* and *Proteus mirabilis*. All other patients achieved primary closure of the surgical incisions, with the exception of two cases that required a split thickness skin graft over the dorsal side of the hand. Regarding secondary surgical outcomes, unplanned reoperation beyond the planned second-stage closure was required in one patient (2.6%), a 47-year-old male with myocardial infarction history and type II diabetes mellitus who required five total surgical interventions (four debridements plus closure) and experienced the longest LOS in the cohort (17 days); one patient additionally required an extra debridement totaling three interventions (2.6%); amputation was performed in one patient (2.6%) due to polymicrobial infection recurrence (*Escherichia coli* and *Proteus mirabilis*) after final closure; and skin grafting was required for soft tissue coverage in two patients (5.3%). Tendon rupture was identified intraoperatively in three patients (7.9%). No patient developed deep-space necrotizing fasciitis or osteomyelitis during the index admission.

The number of outpatient follow-up visits varied widely across the cohort. The mean number of outpatient visits was 2.5 ± 1.9, with a range from 0 to 7 visits. A total of four patients (10.5%) required no outpatient follow-up visits, while 10 patients (26.3%) had one visit. The majority of patients required two outpatient visits, recorded in 15 patients (39.5%). Three visits were documented in five patients (13.2%), whereas four or more visits were required in four patients (10.5%), including seven visits in four cases.

## 4. Discussion

The present study was designed to move beyond the traditional descriptive approach to septic tenosynovitis and to specifically interrogate which factors meaningfully contribute to prolonged hospitalization, using LOS as a clinically relevant and system-oriented outcome. In this context, the observed mean LOS of 7.5 days, with more than 70% of patients requiring hospitalization beyond six days, shows the substantial inpatient resource utilization associated with septic tenosynovitis in a tertiary referral setting [8,19]. An essential conceptual framing must be established at the outset; LOS is an organizational outcome, not a purely clinical one. Duration of hospitalization is shaped by a composite of infection severity, host biology, therapeutic response, and healthcare system factors, including institutional discharge protocols, availability of ambulatory intravenous antibiotic services, and clinician risk tolerance for early discharge in vulnerable patients. This study therefore evaluates predictors of inpatient resource utilization and does not make direct claims about clinical severity, functional prognosis, or long-term outcomes. These distinctions are critical for accurate interpretation of the findings presented below.

Excessive inpatient days are increasingly recognized as clinically relevant beyond their economic implications. Prolonged hospitalization may increase patient exposure to hospital-related complications, including healthcare-associated infections and in-hospital adverse events, while simultaneously generating unnecessary financial costs for both patients and healthcare systems. Beyond these factors, extended LOS may negatively impact patient well-being through reduced mobility, psychological stress, and delayed return to daily activities, while also placing additional strain on healthcare teams through increased workload, resource utilization, and staff fatigue [20].

Within this framework, the most striking finding of the present study was the consistent association between patient-related vulnerability and prolonged hospitalization. All patients with documented comorbidities experienced an LOS ≥ 6 days, a relationship that remained statistically significant (*p* = 0.0026). This observation aligns with existing literature identifying diabetes, vascular disease, and chronic systemic illness as contributors to complicated hand infections and impaired local immune response [21,22,23,24]. Importantly, the uniformity of prolonged LOS in this subgroup suggests that comorbidities act not merely as modifiers of infection severity, but as reliable amplifiers of inpatient care requirements once infection is established [25]. Disaggregation of the comorbidity variable reveals that diabetes mellitus (type I or II) was the most prevalent comorbidity, present in eight of 14 (57.1%) comorbid patients. This finding is consistent with the well-established role of diabetes in potentiating hand infections; hyperglycemia impairs neutrophil function, reduces bactericidal capacity, and delays wound healing, collectively prolonging the inpatient course beyond what would be anticipated from the infection alone [21,22,23,24]. Major cardiovascular or multisystem disease was present in three comorbid patients (21.4%), and chronic renal failure in two (14.3%). Although the small subgroup sizes preclude formal statistical comparisons between comorbidity types, the 100% rate of prolonged LOS regardless of subtype suggests that systemic vulnerability, irrespective of its specific etiology, is the dominant driver of extended hospitalization in this setting.

A similar pattern was observed with respect to immunosuppressive status (*p* = 0.0378). All immunosuppressed patients in the present cohort required prolonged hospitalization, reinforcing the concept that altered host immune response plays a central role in determining inpatient course [26,27]. Notably, immunosuppression emerged as a potential determinant of LOS independent of chronological age, suggesting that biological vulnerability may carry more prognostic weight than age alone. Similarly, neither age nor sex demonstrated a significant association with prolonged hospitalization. While older age has been linked to increased complication rates in hand infections, the present findings suggest that age alone does not reliably predict inpatient duration when considered independently [9]. It is important to acknowledge that immunosuppressive status is a non-modifiable risk factor in the acute setting: it cannot be changed by adjusting antibiotic regimens or surgical timing. Its clinical utility lies primarily in risk stratification. Identification of immunosuppressed patients at admission enables anticipatory care planning with concrete implications: earlier discussion of discharge barriers, proactive social work involvement, preparation of outpatient intravenous antibiotic infrastructure, and calibrated expectation-setting regarding inpatient duration for patients and families. Recognizing immunosuppression as a predictor of prolonged LOS thus carries meaningful practical value for care pathway optimization, even if the risk factor itself cannot be modified acutely. In contrast, several factors traditionally reported in the literature did not independently translate into prolonged hospitalization in the present analysis. Although delayed presentation has been repeatedly associated with worse outcomes in septic tenosynovitis, the association between time to presentation and LOS ≥ 6 days did not reach statistical significance (*p* = 0.1596). This may reflect the relatively small cohort size and the dichotomization of presentation timing, but it may also suggest that once surgical intervention is initiated, inpatient course becomes more strongly influenced by host factors and treatment response than by delay alone [9]. Several interpretations merit consideration. First, the limited statistical power of this 38-patient cohort may have been insufficient to detect a true effect, given that the delayed-presentation subgroup (*n* = 18, delay >7 days) showed a numerically higher rate of prolonged LOS (83.3%) compared to the early group (*n* = 20, 60.0%), suggesting a clinically meaningful trend that may reach significance in larger studies. Second, the presentation timing may have obscured a dose–response relationship between delay duration and inpatient burden. Third, the institutional protocol of aggressive surgical debridement on admission regardless of delay may have attenuated the prognostic impact of timing on LOS. Critically, these findings must not be interpreted as evidence that presentation delay is clinically inconsequential: the established literature consistently demonstrates that earlier intervention is associated with better functional outcomes, lower complication rates, and reduced amputation risk [9,19]. The present analysis evaluates LOS as an organizational endpoint and cannot speak to these clinical outcomes.

Microbiological findings also failed to independently predict prolonged LOS. Patients with positive cultures and those with negative cultures exhibited comparable hospitalization patterns, a finding consistent with prior reports highlighting high rates of culture-negative infections in hand infection. In hand tenosynovitis, negative cultures are not uncommon and do not exclude an underlying infectious process. Prior antibiotic exposure can suppress bacterial growth below the detection threshold of standard cultures, while slow-growing or atypical pathogens, such as nontuberculous mycobacteria or fungi, may require specialized media and prolonged incubation. In addition, deep-seated infections with biofilm formation can limit organism recovery, often leading to persistent symptoms despite initially negative microbiological results [28,29].

When contextualized within the existing literature, the present findings offer a complementary perspective to prior large series. Mamane et al. reported a mean hospital stay of 17 days in a cohort of 120 patients with infectious flexor tenosynovitis, with a wide range extending up to 80 days. Although the mean LOS in the present cohort was substantially shorter, the high proportion of patients requiring hospitalization beyond six days reveals that there is persistent inpatient burden associated with septic tenosynovitis, even in contemporary care settings. Differences in patient selection, institutional protocols, and discharge thresholds may account for these discrepancies [1].

Finally, socioeconomic vulnerability, healthcare disparities, and medical complexity may further modulate discharge timing and contribute to prolonged hospitalization beyond clinical necessity. Patients with multimorbidity or limited access to post-discharge support may be particularly susceptible to delayed discharge, reinforcing the importance of incorporating social and systemic considerations into the management of septic tenosynovitis [30,31]. It is important to explicitly acknowledge that LOS is not a purely biological endpoint: discharge timing is shaped by a composite of infection severity, host vulnerability, therapeutic response, and healthcare system factors. These include the availability of outpatient intravenous antibiotic services, the feasibility of ambulatory wound care, and the patient’s home support network. An immunosuppressed patient, for example, may remain hospitalized longer not only because of a protracted clinical course, but because clinicians prefer closer monitoring in the absence of robust outpatient infrastructure, independent of the actual clinical trajectory. This creates an inherent commingling between clinical and organizational determinants of LOS that cannot be fully disentangled in a retrospective single-center study, and that should be explicitly acknowledged when interpreting the present findings. Future prospective studies should attempt to systematically document discharge-delaying factors (social, logistic, and clinical) to better disentangle these determinants.

In addition to inpatient resource utilization, septic tenosynovitis is associated with a substantial outpatient care burden. In the present cohort, patients required multiple post-discharge follow-up visits, reflecting ongoing needs for wound surveillance, dressing changes, antibiotic monitoring, and functional assessment [32]. Repeated outpatient visits may impose logistical and financial burden on patients, particularly those with limited access to healthcare services, while also increasing demands on outpatient clinics and healthcare personnel. This cumulative burden underscores that the impact of septic tenosynovitis extends beyond the index hospitalization and should be considered when evaluating overall treatment cost and healthcare resource utilization [33,34].

Future research in hand infections should increasingly shift toward prospective, risk-stratified care models that integrate patient-related vulnerability alongside traditional clinical and anatomical factors. At least, multicenter national prospective studies are needed to validate length of stay as a surrogate marker of treatment burden and to determine whether early identification of high-risk patients, such as those with significant comorbidities or immunosuppression, can facilitate tailored perioperative pathways and earlier safe discharge. Additionally, standardized reporting of complications, antibiotic exposure, and functional outcomes would allow more meaningful comparisons across institutions and treatment strategies. Emerging evidence also supports the role of optimized diagnostic algorithms and protocol-driven management in reducing variability of care and improving outcomes in acute hand infections, highlighting the need for consensus-based pathways that balance timely intervention with efficient resource utilization [35,36].

This study is limited by its retrospective design and single-center setting, which may introduce selection bias and restrict generalizability. We acknowledge that analyzing LOS as a continuous variable via linear regression would further strengthen the analytical depth; we propose this as a priority for future prospective studies with larger cohorts. Data on inpatient complications, long-term outcomes, and prior antibiotic exposure before admission were not systematically collected, potentially influencing interpretation of length of stay and microbiological findings. Additionally, institutional discharge practices may have affected hospitalization duration. A further important limitation is the absence of a structured and validated assessment of local infection severity; no formal grading of ischemia, tendon necrosis, purulence degree, or diffuse hand involvement was systematically recorded, meaning that patients with substantially different degrees of anatomical severity were analyzed as a single group. This limits the ability to determine whether infection severity independently contributed to LOS and should be addressed in future studies through adoption of validated scoring systems for hand infections. Additionally, the relatively small cohort size (*n* = 38) limits statistical power, particularly for the immunosuppression subgroup analysis (*n* = 9), and the absence of a control group or multicenter validation restricts the generalizability of the findings. The observed association between sex and LOS (males 82.6% LOS ≥ 6 vs. females 53.3%, *p* = 0.0733) represents an exploratory finding that warrants investigation in larger prospective studies. Future prospective, multicenter studies with standardized outcome reporting are warranted.

## 5. Conclusions

This study demonstrates that, in this single-center cohort, prolonged hospitalization in patients with septic tenosynovitis of the hand is associated predominantly with patient-related systemic vulnerability, particularly the presence of comorbidities (*p* = 0.0026) and immunosuppressive status (*p* = 0.0378), rather than with anatomical involvement, microbiological profile, or delay in presentation. Length of stay emerges as a pragmatic surrogate marker of treatment burden and inpatient resource utilization in this patient population. These findings must be interpreted with appropriate caution: LOS is an organizational endpoint reflecting a combination of biological vulnerability and healthcare system factors, and the present findings do not negate the well-established prognostic importance of early surgical intervention and infection severity in determining clinical outcomes such as functional recovery, amputation risk, and reoperation rates. This study evaluates inpatient resource utilization, not clinical prognosis, and conclusions should be framed accordingly.

These findings suggest that early identification of high-risk patients may allow for improved inpatient planning, optimized perioperative management, and more efficient allocation of healthcare resources. Additionally, the substantial outpatient follow-up burden observed highlights that the impact of septic tenosynovitis extends beyond the index hospitalization.

Future prospective, multicenter studies are warranted to validate these findings and to explore whether targeted care options for vulnerable patient groups can reduce hospitalization duration while maintaining favorable clinical outcomes.

## Figures and Tables

**Table 1:** Mechanism of injury in patients with septic tenosynovitis (ordered by frequency).

Mechanism of Injury	*n*	%
Sharp lacerations	15	39.5
Knife	7	18.4
Metal objects	2	5.3
Glass	2	5.3
Unspecified sharp objects	2	5.3
Cans	1	2.6
Automotive components	1	2.6
Bite-related injuries	9	21.1
Dog bite	4	10.5
Cat bite	3	7.9
Human bite	2	5.3
Puncture wounds	6	15.8
Unknown mechanism	3	7.9
Foreign body retention	2	5.3
Blunt trauma (fall)	1	2.6
Burn	1	2.6
Complex bread machine cut–crush	1	2.6

**Table 2:** Factors associated with LOS of ≥6 days in patients with septic tenosynovitis.

Variable	Category	Los ≥ 6 Days *n* (%)	Los < 6 Days *n* (%)	*p*-Value (Fisher Exact)
Comorbidities	Present (*n* = 14)	14 (100)	0 (0)	0.0026
	Absent (*n* = 24)	13 (54.2)	11 (45.8)	
Immunosuppressive status	Immunosuppressed (*n* = 9)	9 (100)	0 (0)	0.0378
	Not immunosuppressed (*n* = 29)	18 (62.1)	11 (37.9)	
Time to presentation	Early (*n* = 20)	12 (60.0)	8 (40.0)	0.1596
	Delayed (*n* = 18)	15 (83.3)	3 (16.7)	
Age	<65 years (*n* = 30)	21 (70.0)	9 (30.0)	1.0
	≥65 years (*n* = 8)	6 (75.0)	2 (25.0)	
Microbiological culture	Positive (*n* = 21)	16 (76.2)	5 (23.8)	0.491
	Negative (*n* = 17)	11 (64.7)	6 (35.3)	

**Table 3:** Microbiological isolates identified in septic tenosynovitis.

Microorganism	*n* (26)	%
*Staphylococcus* spp.	14	53.8
*Staphylococcus aureus* (MSSA)	7	26.9
*Staphylococcus aureus* (MRSA)	2	7.7
*Staphylococcus epidermidis*	2	7.7
*Staphylococcus hominis*	2	7.7
Coagulase-negative *Staphylococcus* (unspecified)	1	3.8
*Streptococcus* spp.	3	11.5
*Streptococcus pyogenes*	1	3.8
β-hemolytic *Streptococcus* group A	1	3.8
*Streptococcus* group D	1	3.8
Gram-negative bacilli	9	34.6
* Proteus mirabilis*	2	7.7
* Escherichia coli*	1	3.8
* Klebsiella pneumoniae*	1	3.8
* Klebsiella oxytoca*	1	3.8
* Enterobacter cloacae*	1	3.8
* Citrobacter freundii*	1	3.8
* Pasteurella multocida*	1	3.8

## Data Availability

The dataset is available on request from the authors.

## Abbreviations

The following abbreviations are used in this manuscript:

LOS	Length of stay
MDR	Multi-drug resistant
MSSA	Methicillin-susceptible *Staphylococcus aureus*
MRSA	Methicillin-resistant *Staphylococcus aureus*
